# Aberrant intra- and inter-network connectivity architectures in Alzheimer’s disease and mild cognitive impairment

**DOI:** 10.1038/srep14824

**Published:** 2015-10-06

**Authors:** Pan Wang, Bo Zhou, Hongxiang Yao, Yafeng Zhan, Zengqiang Zhang, Yue Cui, Kaibin Xu, Jianhua Ma, Luning Wang, Ningyu An, Xi Zhang, Yong Liu, Tianzi Jiang

**Affiliations:** 1 Department of Neurology, Institute of Geriatrics and Gerontology, Chinese PLA General Hospital, Beijing, 100853, China; 2 Department of Neurology, Tianjin Huanhu Hospital, Tianjin, 300060, China; 3 Department of Radiology, Chinese PLA General Hospital, Beijing, 100853, China; 4 Brainnetome Center, Institute of Automation, Chinese Academy of Sciences, Beijing, 100190, China; 5 School of Biomedical Engineering, Southern Medical University, Guangzhou, Guangdong, 510515, China; 6 Hainan Branch of Chinese PLA General Hospital, Sanya, 572014, China; 7 National Laboratory of Pattern Recognition, Institute of Automation, Chinese Academy of Sciences, Beijing, 100190, China; 8 CAS Center for Excellence in Brain Science, Institute of Automation, Chinese Academy of Sciences, Beijing 100190, China

## Abstract

Alzheimer’s disease (AD) patients and those with high-risk mild cognitive impairment are increasingly considered to have dysfunction syndromes. Large-scale network studies based on neuroimaging techniques may provide additional insight into AD pathophysiology. The aim of the present study is to evaluate the impaired network functional connectivity with the disease progression. For this purpose, we explored altered functional connectivities based on previously well-defined brain areas that comprise the five key functional systems [the default mode network (DMN), dorsal attention network (DAN), control network (CON), salience network (SAL), sensorimotor network (SMN)] in 35 with AD and 27 with mild cognitive impairment (MCI) subjects, compared with 27 normal cognitive subjects. Based on three levels of analysis, we found that intra- and inter-network connectivity were impaired in AD. Importantly, the interaction between the sensorimotor and attention functions was first attacked at the MCI stage and then extended to the key functional systems in the AD individuals. Lower cognitive ability (lower MMSE scores) was significantly associated with greater reductions in intra- and inter-network connectivity across all patient groups. These profiles indicate that aberrant intra- and inter-network dysfunctions might be potential biomarkers or predictors of AD progression and provide new insight into AD pathophysiology.

Alzheimer’s disease (AD) is an age-related neurological degenerative disorder and is the most common cause of dementia in older subjects. Clinically, AD typically begins with episodic memory impairment, followed by slow progression toward more general impairment in daily activities, such as attention, executive function, language, and visuospatial functions, ultimately leading to a loss of independent daily living and causing a heavy toll on the patient and the family. Mild cognitive impairment (MCI), especially amnestic MCI (aMCI), has been considered the prodromal stage of dementia and has a high risk of converting to AD^1^,^2^,^3^,^4^.

The accumulation of amyloid beta (Aβ) plaques and tau tangles, as early markers for AD, is found widely in the disease progression of aMCI/AD^5^,^6^. Additionally, convergence evidence has confirmed AD subjects were associated with gray matter atrophy or volume reduction^7^ and thinner cortices^8^,^9^ based on structural magnetic resonance imaging (MRI). Functional imaging may bridge the finding between early molecular alterations (for example, amyloid beta deposition) and later clinical symptoms (e.g., cognitive changes) or structural changes in AD^10^. Resting-state functional MRI (fMRI) connectivity is a noninvasive measure that enables the assessment of the temporal correlations in spontaneous brain activity^11^,^12^.The correlational structures of these fluctuations among spatially distributed brain regions are used to identify connectivities and networks within the brain, and the resting-state networks (RSNs) are characterized by spatially coherent spontaneous activities; they are also used to depict the connectivity patterns that are commonly involved in functions such as the sensory, attention, and executive control networks and default mode processing^13^,^14^.

The research on large-scale RSNs offers a typical paradigm for investigating the relationship between cognitive dysfunction and brain activity alterations in neurological disorders^15^,^16^,^17^. In terms of AD/MCI, convergence evidence based on fMRI studies showed that functional integration is disturbed and that the functional changes are related to cognitive variables in the resting-state. For example, recent studies have demonstrated that functional connectivity and networks were selectively impaired in individuals with AD or at risk for AD, especially in higher cognitive RSNs such as the default mode network (DMN)^18^,^19^,^20^,^21^, attention network^22^,^23^, executive control network^24^ and salience network(SAL)^25^,^26^. All of these findings support the idea that AD is a disconnection syndrome^27^,^28^ and that the selective impairment patterns in the RSNs may characterize the traits and states of AD^18^. However, most of these studies mainly focused on the connectivity alterations within individual networks or between one or two RSNs, which may limit our understanding of the pathophysiological substrate of AD. Fortunately, researchers began to realize the importance of studying the functional disturbances from an integrative perspective. For example, Wang and colleagues studied the connectivity patterns among ten modes based on a previously defined template and demonstrated that nearly half of the decreased functional connectivity was between the prefrontal and parietal lobes^29^. Li and colleagues found that the network-to-network connectivity patterns changed among eight cognitive networks and sensory networks in AD patients^30^, and some network studies have demonstrated the brain’s topologically reorganized architecture in AD and MCI^31^,^32^,^33^,^34^,^35^. Importantly, the intra-network and inter-network patterns of the resting-state connections in subjects with AD and autosomal dominant AD and individuals at high risk (family history, APOE-4 allele and amyloid burden) for AD have been well studied, and the results have demonstrated that the large-scale brain networks are vulnerable in AD patients and in individuals at high risk of converting to AD^25^,^36^,^37^,^38^,^39^,^40^,^41^,^42^.

Inspired by the above studies, we speculated that intra-network functional connectivity would be impaired in AD and MCI in comparison with normal cognitive (NC) healthy volunteers and that the interactions between different RSNs would change simultaneously. We also expected that brain function connectivities would differ based on patient variations in cognitive impairment severity. To test these hypotheses, we investigated the altered functional connectivity patterns at three levels (integrity, network and connectivity) based on five previously defined key RSNs: the default mode network (DMN), dorsal attention network (DAN), control network (CON), salience network (SAL) and sensorimotor network (SMN), based on the fMRI data acquired under no-task conditions from 35 patients with AD, 27 patients with MCI and 27 age-matched NC volunteers. In addition, we also explored the relationships between the Mini Mental State Examination (MMSE) scores and these strengths of functional connectivities at each scale by using Pearson’s correlations after controlling for age and gender effects in the patient groups.

## Materials and Methods

### Ethics statement

This study was approved by the Medical Ethics Committee of the Chinese PLA General Hospital. All participants were assessed at the Chinese PLA General Hospital, and written consent forms were obtained from the subjects or their legal guardians (spouses or children) and the methods were carried out in accordance with the approved guidelines.

A portion of the subjects were included in our previous studies on the perceptual and response interference in AD^43^ and the changes in regional brain activity^44^, the functional connectivity patterns of the amygdala^45^, and the same dataset was used in studying the functional connectivity patterns of the thalamic^46^ and marginal divisions^47^ in the resting-state and also the impaired gray matter volume pattern^48^ based on structural MRI. Here, we provide a brief introduction regarding the data inclusion and exclusion criteria, acquisition and processing to maintain the scientific integrity of the present study. Detailed data descriptions can be found in Part I of the supplemental material.

### Subjects

The subjects were recruited from two sources: as outpatients from the Chinese PLA General Hospital or through a website advertisement (http://www.301ad.com.cn, Chinese version). All subjects met the identical methodological stringency criteria, and comprehensive clinical details were described in our previous studies; additional details regarding participant selection and exclusion for this data set can be found elsewhere^44^,^45^,^46^,^47^ and Part I of the supplemental material. Briefly, the recruited AD patients were diagnosed using the National Institute of Neurological and Communicative Disorders and Stroke and the Alzheimer’s Disease and Related Disorders Association criteria for probable AD. The enrolled MCI patients fulfilled the diagnostic criteria described by Petersen *et al.*^2^. At the same time, our AD and MCI patients also met the core clinical criteria of the new diagnostic criteria for probable AD dementia and MCI due to AD^49^,^50^. Exclusion criteria included significant neurological or psychiatric illness that can influence cognitive functions as well as significant unstable systemic illness or organ failure. In addition, patients with a metallic foreign body were also excluded from the study for security and imaging quality control reasons. No subjects were treated with any medication that could have influenced their cognition during the data collection. Each subject was right-handed and underwent a battery of neuropsychological tests: the Mini-Mental State Examination (MMSE), Auditory Verbal Learning Test (AVLT), Geriatric Depression Scale, Clinical Dementia Rating (CDR) and Activities of Daily Living (ADL) scale.

Briefly, after excluding subjects with large head motions (see the criteria in data preprocessing), 89 subjects—35 AD patients, 27 MCI subjects, and 27 age- and gender-matched NC subjects—were included for further analysis. Demographic and neuropsychological details for the subjects are shown in Table 1 and can be found in our previous studies^44^,^45^,^46^,^47^.

### fMRI data acquisition and preprocessing

As was previous noted, the MRI scans were performed at the Chinese PLA General Hospital, Beijing, China, with a 3.0 T GE MR system (GE Healthcare, USA) using a standard head coil. During the scanning, the subjects were instructed to keep their eyes closed and relax; comfortable foam padding was used to minimize head motion, and ear plugs were used to reduce the scanner noise. Before the resting fMRI data were collected, T2-weighted images were collected and evaluated by two senior radiologists. Resting-state fMRI data were acquired using an echo planar imaging (EPI) sequence with repetition time = 2000 ms, echo time = 30 ms, flip angle = 90°, matrix = 64 × 64, field of view = 220 mm × 220 mm, slice thickness = 3 mm and slice gap = 1 mm. Each volume was composed of 30 axial slices, and each functional run lasted for 6 minutes and 40 seconds.

The data were preprocessed using the same steps as those in our previous studies using the in-house Brainnetome fMRI toolkit (Brat, www.brainnetome.org/brat) based on statistical parametric mapping (SPM8, http://www.fil.ion.ucl.ac.uk/spm). These steps were: (1) slice-timing with reference slice = 2, (2) realignment to the first volume, (3) normalization to a standard EPI template and reslicing to 2 × 2 × 2 mm cubic voxels, (4) de-noising by regressing out multiple effects, i.e., the six motion parameters, the constant, the linear drift and the mean time series of all voxels within the white matter and cerebrospinal fluid, and (5) temporal filtering (0.01–0.08 Hz) to reduce noise. The data were not further smoothed for we intended to investigate the connectivity patterns of the prior defined seed regions. Group differences in head motion followed those in Van Dijk’s study^51^, and no significant differences in head motion were found among the three groups (Table 1)^47^.

### Connectivity analysis

The five RSNs used in this study have been well investigated in previous studies^36^,^37^,^38^,^39^,^52^. To maintain consistency with these prior studies, the seed regions in the present study were defined a prior based on a study by Dr. Ances; specifically, thirty-six spherical (6 mm radius) regions of interest (ROIs) that represented the five RSNs(DMN, DAN, SAL, CON, and SMN) were obtained using the Brainnetome fMRI toolkit. As in previous studies^36^,^37^,^38^,^39^,^52^, the SMN included the primary auditory, primary visual, and somatomotor cortices. The Montreal Neurological Institute coordinates of the 36 ROIs are presented in Table S1, and the individual ROIs that were displayed on the brain surfaces are shown in Figure S1 in the supplemental material.

The representing mean time series was estimated by averaging the time series of all voxels in this ROI. The Pearson’s correlation coefficients were computed between each pair of ROIs for each subject. Fisher’s r-to-z transformation was applied to obtain Z scores and improve the normality of the correlation coefficients. Then, the connectivity pattern of these ROIs was investigated at the following three levels:

1. Integrity: for each node, the integrity Z score was defined as the sum of connection strength, that is, 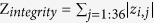, where 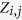 is the Z score between the i^th^ and j^th^ ROI. This measure is equivalent to “degree centrality” in the graph theory.
2. Network: for each of the five RSNs, the intra-network strength was defined as the mean connection strength of the ROIs in the same network, i.e., 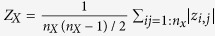, where 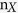 is the number of ROIs within a specific subnetwork X and k is a range from 1 to 5 that represents the five subnetworks. For each pair of subnetworks, the inter-network connectivity strength was defined as the mean strength of all of the possible connections, that is, 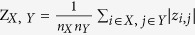, where X and Y represent the subnetworks of the five selected RSNs.
3. Connectivity: the Z scores of each pair of ROIs among the included 36 seed regions.

### Statistical analysis

To assess the statistical significance, the Z scores at each level were entered into one-way analyses of variance (ANOVAs) with group as a factor (3 groups: NC, MCI, AD) after age and gender effects were regressed out using a general linear model; the statistical significance was P < 0.05. Considering that we have performed many times comparisons among the three groups, we performed a 10,000 times random permutations to test if the identified altered connections are really significant. For all the identified impaired connectivity, the significant effects were assessed by post hoc two-sample, two-sided t-tests of NC versus MCI, NC versus AD and MCI versus AD (P < 0.05).

To investigate the relationship between functional connectivity and cognitive ability, we also explored the Pearson’s correlations between the MMSE scores and the Z scores at each level in the MCI, AD and MCI plus AD groups. Because these relationships were exploratory in nature, we used a statistical significance level of P < 0.05 (uncorrected).

## Results

### Group differences at the three levels

For each group, a 36 × 36 functional connectivity matrix was computed. In the NC group, the majority of strong positive functional connectivities were within each network, and most negative correlations were between different networks (Figure S2A–C). A similar pattern was observed in the MCI and AD groups, except that the intra-network and inter-network correlations were decreased (Figure S2D–F).

**Figure 1:**
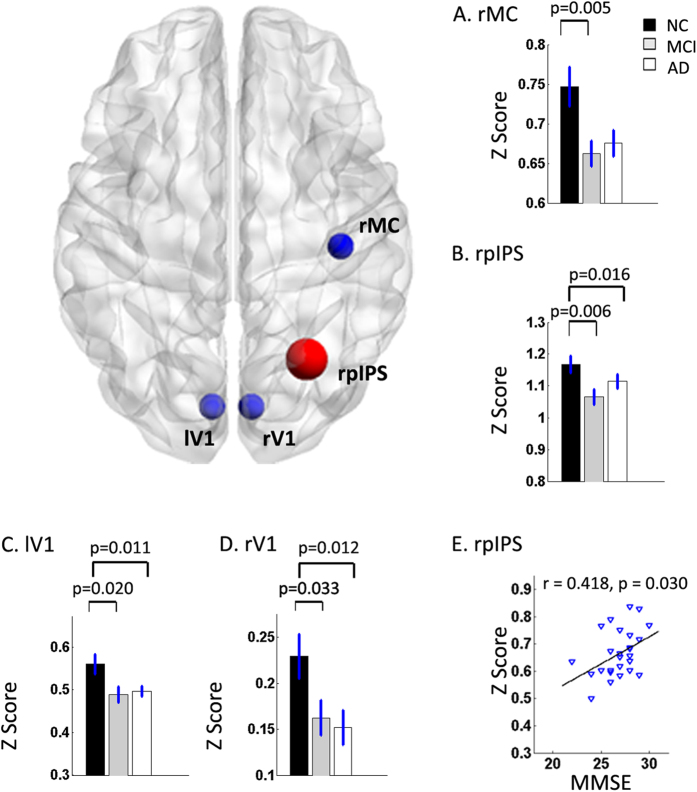
Group effects by one-way ANOVA for and the relationship between integrity connectivity and MMSE scores. Bar graphs show the differences in the mean Z scores for the affected regions among the three groups. The changed regions were anchored in the right motor cortex (rMC) (**A**), the right posterior intraparietal sulcus (rpIPS) (**B**) and the bilateral primary visual (V1) (**C**,**D**). The Z scores for the bilateral V1 and rpIPS were higher in the NC group than in the AD and MCI groups, and the rMC of the NC group was greater than that of the MCI group only. The NC group is indicated by black rectangles, the MCI group by grey rectangles and the AD group by white rectangles. The error bars represent the standard error of each subgroup. The scatter plots show the relationship between the mean Z scores of rpIPS and MMSE scores in the MCI patients (r = −0.418, p = 0.03) (**E**).

At the integrity level, significant group differences were anchored in the right motor cortex (rMC), the bilateral primary visual (V1), and the right posterior intraparietal sulcus (rpIPS) (Table 2, Fig. 1). Post hoc analysis showed that the integrity connectivity of these regions was reduced in the AD and MCI groups compared with the NC, but there was no significant alteration between the AD and MCI groups (Fig. 1).

**Figure 2:**
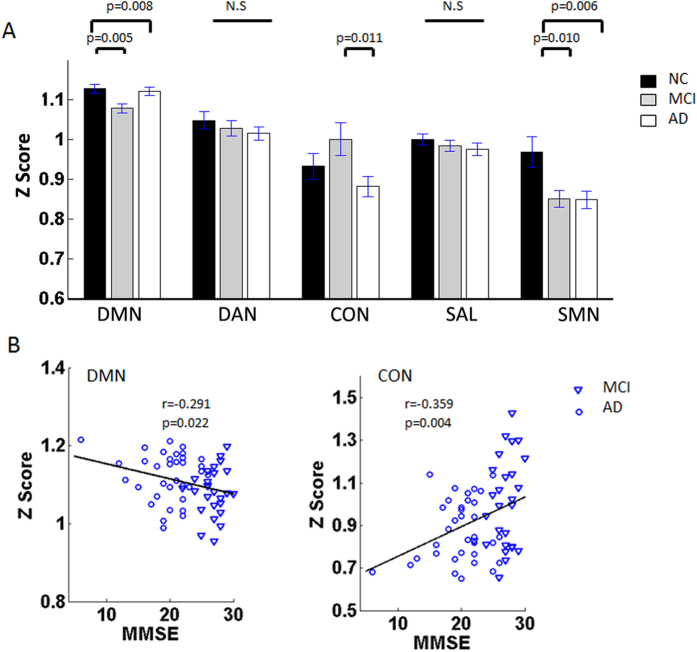
(**A**) The differences in mean Z scores within the five RSNs. The bar graph shows that the majority of network composite Z scores tended to lower value in DMN and SMN with increasing disease severity. Compared with the NC group, the Z scores within the DMN and SMN showed significant decreases in the AD and MCI groups (DMN, p = 0.005 for MCI, p = 0.008 for AD; and SMN, p = 0.01 for MCI, p = 0.006 for AD). A transient increase in composite Z scores was seen in the CON between NC and MCI, but a sharp decrease (MCI versus AD, p = 0.011) occurred in the AD group. The NC group is indicated by black rectangles, the MCI group by grey rectangles and the AD group by white rectangles. The error bars represent the standard error of each subgroup. (**B**) Scatter plots showing the relationship between the mean Z scores for the DMN (r = −0.291, p = 0.022), the CON (r = −0.359, p = 0.004) and the MMSE scores in the AD and MCI patients. MCI patients are indicated by the blue triangles and the AD patients by the blue circles.

**Figure 3:**
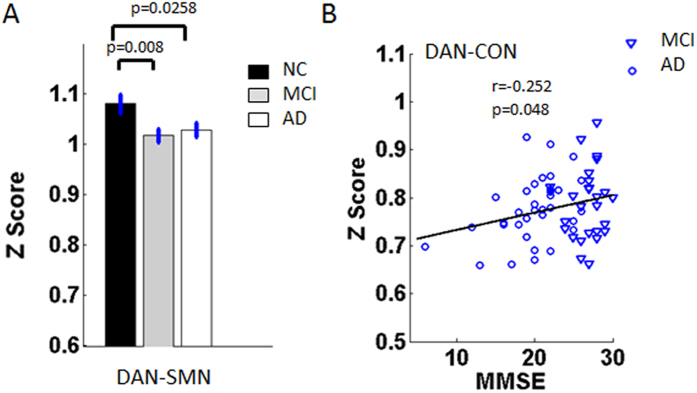
(**A**) Bar graph of the DAN-SAN connectivity strength in the three groups, which shows that the strength differed significantly in the NC group compared with the MCI (p = 0.008) and AD (p = 0.0258) groups. The NC group is indicated by black rectangles, the MCI group by grey rectangles and the AD group by white rectangles. The error bars represent the standard error of each subgroup. (**B**) Scatter plots showing the relationship between the mean Z scores for DAN-CON (r = −0.252, p = 0.048) and the MMSE scores in the AD and MCI patients. The MCI patients are indicated by the blue triangles and the AD patients by the blue circles.

At the network level, the composite Z scores of the DMN, CON and SMN showed significant differences in the severity of the cognitive impairments among the groups (Table 3, Fig. 2A). Compared with those for the NC group, the Z scores within the DMN and SMN showed significant decreases in the AD and MCI groups (Fig. 2A). Particularly, the Z scores for CON showed a slight increase in the MCI group but a sharp decrease in the AD group (Fig. 2A). For the inter-network pairs, only the DAN-SMN connectivity showed a significant decrease in the AD and MCI groups (Table 4, Fig. 3A).

**Figure 4:**
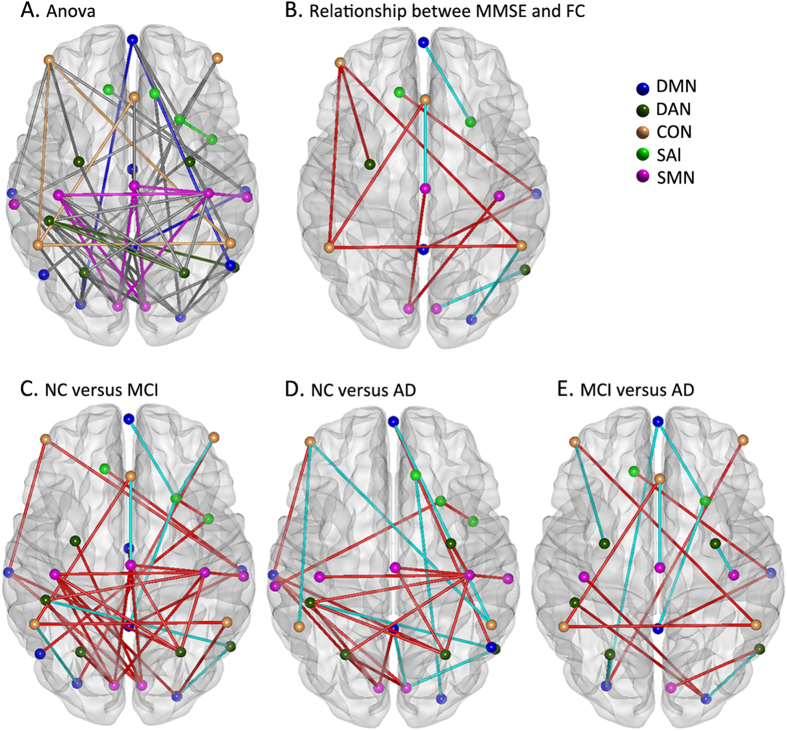
(**A**) Distribution of the altered functional connectivity. All affected ROI pairs for all 5 RSNs (blue for DMN, dark green for DAN, yellow for CON, green for SAL and pink for SMN), except for intra-network ROI pairs, were mainly distributed between the DMN and other RSNs and between the DAN and the SMN.(**B**) The correlations between the functional strength of the affected ROI pairs and the MMSE scores. The blue color represents the functional connectivity that shows positive correlations with the MMSE scores, and the red color represents negative correlations. (**C**) The differences in connectivity between the NC and MCI groups. (**D**) The differences in connectivity between the NC and AD groups. (**E**) The differences in connectivity between the MCI and AD groups. In the subfigure (**C**–**E**), the blue color indicates that the functional connectivity of the former group is stronger than that for the latter, and the red color indicates the reverse. For details, please refer to Table S2 in the supplemental material.

As Fig. 4 shows, a large number of changed intra-network and inter-network functional connectivities were identified by one-way ANOVA after age and gender effects were controlled using linear regress (Fig. 4A; details of the statistical values can be found in Table S2). The most significantly affected pairs were mainly distributed between the DMN and other RSNs and between the DAN and SMN (Fig. 4A). Post hoc analysis strengthened these manifestations (Fig. 4C–E). Specifically, the most significant alterations were mainly distributed between the DAN and the SMN in the MCI subjects in comparison with the NC individuals (Fig. 4C).

For all the identified impaired connectivities, random permutation tests indicate that these findings are significant with P < 0.05 (Figs 1, 2, 3, 4. Tables 2, 3, 4, S2).

### Correlations between altered connectivity and MMSE scores

At the integrity level, the connectivities of the laPFC and the rpIPS demonstrated significant correlations with the MMSE scores in the MCI patients (Table 2, Fig. 1). At the network level, the affected intra-network interactions between the DMN and CON and the DAN and CON were significantly correlated with the MMSE scores in the AD and MCI patients (Tables 3, 4, Figs 2B and 3B). Our results also demonstrated that 9 connectivity pairs showed positive correlations with MMSE scores and that 4 connectivity pairs showed negative correlations with MMSE scores in the identified impaired connectivity pairs (Fig. 4B; for details, please refer to Table S2).

## Discussion

In the present study, widespread impaired functional connectivity patterns including intra-network and inter-network disconnections that could have been the basis for cognitive impairment were identified in the MCI and AD patients. Based on the integrity-level analysis, the differences were anchored in the right motor cortex (rMC), the bilateral primary visual (V1) and the right posterior intraparietal sulcus (rpIPS) in the AD and MCI subjects (Table 2, Fig. 1). The connectivities within the DMN and SMN were significantly decreased in the MCI and AD groups, and the connectivities within the CON increased slightly in the MCI group whereas it showed a sharp decrease in the AD group (Fig. 2A). Connectivity analysis of both the inter-network and the ROI pairs showed that the DAN-SMN connectivity was impaired significantly in the patient groups, especially in the MCI individuals. Among the identified impaired connectivities, some demonstrated significant correlations with cognitive ability as assessed by the MMSE scores (Fig. 4B and Table S2).

### Impaired intra-network connectivity within the RSNs

Consistent with previous studies, the present study demonstrated that the default mode network (DMN) was one of the most affected network in the AD^21^ and MCI^34^ subjects (Fig. 2A,B). The DMN plays a key role in cognitive processes, especially in episodic memory processing^53^,^54^, and the impaired performance of episodic memory is one of the core features of a diagnosis of AD^6^; this could be one of the main reasons that impaired functional connectivity within the DMN has been frequently identified in AD and MCI patients using multiple imaging techniques^6^,^19^,^36^. In addition, amyloid plaques, which have been considered the main pathophysiological process of AD to date, were found to be preferentially deposited in regions of the DMN^55^ and to cause the impaired resting-state fMRI connectivity^40^,^56^,^57^,^58^. Based on the DMN’s core role in modulating daily cognition, functional deficits in the DMN might contribute to AD pathology and might be potential biomarkers for distinguishing AD from MCI.

In the present study, the functional connectivity within the CON increased slightly in the MCI group, whereas it decreased sharply in the AD group (Fig. 2A). As was described, the CON is crucial for active maintenance of and manipulating information in working memory and for rule-based problem solving and decision making, and it is related to executive function^59^,^60^,^61^. MCI patients showed subtle executive dysfunctions in higher-order activities, such as financial capacity^62^, but everyday abilities were preserved; hence the increased connectivity within the CON may reflect a coherent compensatory recruitment in MCI patients. Consistent with this, neuroimaging studies have suggested that the increased prefrontal activity (the main region of CON) reflects compensatory strategies used in performing cognitive tasks^63^,^64^. Clinically, AD patients lose the ability to manage their daily life activities, which could be because activities such as cooking, shopping, and driving are target-based problem-solving tasks that require the participation of the executive control brain regions^65^ and executive dysfunctions are considered pervasive^66^. Hence, the decreased connectivity within the CON in AD patients might reflect the foundation of executive dysfunction.

An interesting finding was that our results demonstrated that the functional connectivity within the sensorimotor network (SMN) was impaired in the MCI and AD subjects (Figs 1 and 2A). The SMN, composed of the primary visual, auditory and somatomotor cortices, plays a role in receiving external signals, which could aid in perceiving the world, selecting the relevant information and determining the target; it then conveys the signals to the attention or control systems to induce reasonable responses. For many years, the SMN was usually thought to be relatively stable, and was seen as a reference network in studying AD and MCI^39^,^67^. Although a number of studies have indicated that the functional changes in the olfaction, hearing, visual, and motor systems (the major components of the SMN) might precede the onset of cognitive impairments, worsen as the disease progresses, and be strong risk factors for AD^68^,^69^,^70^,^71^, this impaired functional connectivity, together with the recognition that AD pathology will develop over many years, raises the exciting possibility that declines in specific primary daily functions may be early noninvasive biomarkers for AD. Even more provocatively, treating these daily symptoms may help to delay or treat MCI/AD^72^. Our results provided additional evidence that the affected SMN should arouse attention not only as a reference stable system in studying AD and MCI.

### Impaired inter-network connectivity between RSNs in AD and MCI

Resting-state functional connectivity and network analysis provides a new tool for mapping large-scale function and dysfunction in the brain system^12^,^73^.Inter-network connectivity, especially between the DAN and the SMN, was impaired in the AD and MCI groups (Fig. 3A, Fig. 4A,C,D and Table S2), and this phenomenon prompted us to rethink the role of the interactions between the RSNs in understanding AD pathology and clinical performance. The dorsal attention network (DAN) is one of the networks that is associated with cognitive functions, and the multiple somatosensory integrations of sensory, motor and cognitive systems provide the signals for the organism to perceive and respond to its environment; that is, deficits in any of these components will lead to impaired function at the clinical level^66^,^72^,^74^. Often, decreased attention and sensory or motor declines are seen as signs of aging; clinically, the performance of these functions is poor in MCI individuals and even worse in AD subjects^75^,^76^. Our research prompted us to infer that the impaired connectivity within the SMN or reduced connectivity between the SMN and the DAN might be one of the reasons for the attention deficits in the early stages of AD and MCI. Additionally, convergence evidence has suggested that AD pathology develops over many years, raising the exciting possibility that daily cognitive impairments, even very slight (such as reduced sensory or motor ability) may be early, noninvasive markers for AD^72^.

In addition to the above-discussed SMN and DAN, the interactions among the CON, SAL and DMN were also impaired in the AD and MCI groups (Fig. 4A,D,E). Previous resting state fMRI studies have demonstrated aberrant functional connectivity of DMN, SAL and CON in AD^36^,^77^, aMCI^78^ and even in normal cognition with amyloid burden^40^,^79^. Convergent evidence has suggested that these three brain systems were closely correlated and play particularly crucial roles in higher cognitive function^15^,^80^,^81^. Functional connectivity between the SAL and DMN is important for cognitive control^80^,^82^, and the SAL also plays a central role in switching between the CON and the DMN^80^,^82^,^83^,^84^. Our findings provide additional evidence to support the viewpoint that the CON, SAL and DMN are intrinsically well-organized in normal healthy subjects and that aberrations in these networks are the prominent features of functional deficits in AD^16^,^83^,^85^. By contrast, the DMN plays a core role in brain activity and other networks that are involved in succession, which suggests that AD pathology might spread from the DMN to the nearby networks, including those involved in visuospatial and executive function, and in other peripheral networks^86^. Therefore, we speculated that AD patients who show clinical attention deficits, memory impairment, executive dysfunction and other disabilities might be reflecting the integration dysfunction in the different brain networks^31^,^87^.

### Further discussion, limitation and future directions

It should be noted that for multiple metrics but the CON (Figs 2, 3, 4), MCI subjects tend to be similar to AD patients (and different from controls), with little differences between MCI and AD patients. This might because the severity of AD individuals were still mild (35 subjects, 25 with CDR = 1, 10 with CDR = 2) and the variability among patients was relatively high^46^. This might be the reason that we only found some significant correlations between the used measures and cognitive ability in the MCI and AD patients groups (Figs 1, 2, 3 and Table S2). Another possible reason is that the MCI subjects might include different subgroups, for not all the MCI individuals will convert to AD. In fact, for all the used data in the present study, 14 of the 27 MCI subjects were recruited to return for an examination and data collection after around one year (range: 10–16 months), one subject converted to AD^47^. Hence, a long time longitudinal study is needed for further investigation.

Notably, the impaired connectivity at all three levels (integrity, network and connectivity) were significantly correlated with performances on the Mini Mental State Examination (MMSE) neuropsychological tests (Figs 1, 2B, 3B and 4B). The MMSE involves multiple cognitive domains and reflects global brain function; it can be used as a tool for quantitatively assessing the severity of cognitive impairment and reflects the cognitive changes that occur during the progression of AD^88^. As discussed in our previous work, the correlations between MMSE and connectivity and network markers are relatively scant because the MMSE is a brief general screening tool^34^. However, based on the robust findings from previous studies^21^,^36^,^89^, including our own, we speculated that the correlations between MMSE scores and network functional connectivity indicate that abnormal brain function might be a feature that represents disease severity and could potentially be used as an early marker to distinguish patients from healthy subjects.

As is widely known, the whole brain network is complex, varied, and interrelated; the five networks in this study are merely a small part of this complex system, and thus, a whole-brain network analysis with finely defined regions is needed in the future. Second, cross-sectional research cannot dynamically observe changes in network patterns with disease progression^36^. Also many studies have found neuronal dysfunction and disconnection of brain network in normal cognition with amyloid burden^40^,^41^,^42^,^79^ or APOE e4 carrier^90^,^91^. Hence, longitudinal studies combining multiple imaging measures (such as, fMRI, structure MRI, PET etc.) and genetic genotype are needed in the future to follow individuals from healthy to disease states and to different severity levels, exploring network-vulnerability interactions, and to study different subtypes of AD and MCI.

## Conclusion

The novel finding of the present study is the relationship between disease severity and impaired intra-network and inter-network connectivity from global to fine-pair connectivity in AD and MCI patients. The results demonstrated a progressive alteration of network connectivity; at the early stage of the disease (MCI), sensorimotor and attention functions were involved, but with disease progression, the whole-brain function degeneration induced a wider range of inter-network impairments. All of these findings provide new insight into AD pathophysiology and suggest that altered network connectivity patterns may be useful for the preclinical determination of AD.

## Additional Information

**How to cite this article**: Wang, P. *et al.* Aberrant intra- and inter-network connectivity architectures in Alzheimer,s disease and mild cognitive impairment. *Sci. Rep.* **5**, 14824; doi: 10.1038/srep14824 (2015).

## Supplementary Material

Supplementary Information

## Figures and Tables

**Table 1:** Demographic, clinical and neuropsychological data in normal control (NC), mild cognitive impairment (MCI) and Alzheimer’s disease (AD) subjects.

	NC(n = 27)	MCI(n = 27)	AD(n = 35)	p value
Gender (M/F)	16/11	13/14	12/23	0.143
Age (year)	69.2 ± 6.5	73.8 ± 7.8	72.4 ± 8.5	0.090
MMSE	28.9 ± 1.0	26.8 ± 1.8^a^	19.7 ± 4.1^a^,^b^	<0.001
CDR	0	0.5	1.3 ± 0.5^a^,^b^	<0.001
AVLT-Immediate Recall^c^	5.9 ± 1.1	4.6 ± 1.5	2.6 ± 1.6^a^,^b^	<0.001
AVLT-Delay Recall^c^	5.8 ± 2.0	3.1 ± 2.0^a^	0.6 ± 1.2^a^,^b^	<0.001
ApoE e4 carrier (%)^d^	15%	38%	64%	—
Head Motion	0.25 ± 0.27	0.16 ± 0.10	0.30 ± 0.27	0.084

MMSE, mini-mental state examination; CDR, Clinical Dementia Rating; AVLT, auditory verbal learning test, ApoE, apolipoprotein E.

Chi-square was used for gender comparisons, One-way ANOVA with Bonferroni post hoc test was used for age, and neuropsychological tests comparisons.

^a^Significant compared to NC.

^b^Significant compared to MCI.

^c^Three AD subjects refuse to continue this test.

^d^66 (NC = 20, MCI = 21, AD = 25) of the 89 subjects have ApoE genotype.

**Table 2:** Group effects and correlations with MMSE scores at the integrity level.

RSN	ANOVA			Correlation (AD&MCI)	
	F	p	p_permutation	r	p
rpIPS	5.160	0.008	0.006		
rMC	4.391	0.015	0.015		
lV1	4.659	0.012	0.011		
rV1	4.050	0.021	0.019		
laPFC				0.274	0.032

Abbreviation: rpIPS: right anterior intraparietal sulcus; rMC: right motor cortex; lV1: left primary visual cortex; rV1: right primary visual cortex; laPFC: left anterior prefrontal cortex.

**Table 3:** Group effects and correlations with MMSE scores within individual RSNs.

RSN	ANOVA			Correlation (MCI&AD)	
	F	p	p_permutation	r	p
**DMN**	**5.365**	**0.006**	0.006	**−0.291**	**0.022**
DAN	0.767	0.468		0.123	0.343
**CON**	**3.491**	**0.035**	0.033	**0.359**	**0.004**
SAL	0.720	0.490		−0.001	0.995
**SMN**	**5.917**	**0.004**	0.003	0.098	0.451

Abbreviation: DMN: default mode network; DAN: dorsal attention network; CON: control network; SAL: salience network; SMN: sensorimotor network.

**Table 4:** Group effects and correlations with MMSE scores on RSN pairs.

RSN	ANOVA			Correlation (AD&MCI)	
	F	p	p_permutation	r	p
DAN-SMN	4.543	0.013	0.011		
CON-SAL				0.252	0.048

Abbreviation: DMN: default mode network; DAN: dorsal attention network; CON: control network; SAL: salience network; SMN: sensorimotor network.

## References

[b1] Petersen R. C. *et al.* Aging, memory, and mild cognitive impairment. Int Psychogeriatr 9 Suppl 1, 65–69 (1997). 9447429 10.1017/s1041610297004717

[b2] Petersen R. C. *et al.* Mild cognitive impairment: clinical characterization and outcome. Arch Neurol 56, 303–308 (1999). 10190820 10.1001/archneur.56.3.303

[b3] Morris J. C. *et al.* Mild cognitive impairment represents early-stage Alzheimer disease. Arch Neurol 58, 397–405 (2001). 11255443 10.1001/archneur.58.3.397

[b4] Petersen R. C. *et al.* Mild cognitive impairment: ten years later. Arch Neurol 66, 1447–1455 (2009). 20008648 10.1001/archneurol.2009.266 PMC3081688

[b5] Jack C. R. Jr. *et al.* Introduction to the recommendations from the National Institute on Aging-Alzheimer’s Association workgroups on diagnostic guidelines for Alzheimer’s disease. Alzheimers Dement 7, 257–262 (2011). 21514247 10.1016/j.jalz.2011.03.004 PMC3096735

[b6] Dubois B. *et al.* Advancing research diagnostic criteria for Alzheimer’s disease: the IWG-2 criteria. Lancet Neurol 13, 614–629 (2014). 24849862 10.1016/S1474-4422(14)70090-0

[b7] Yang J. *et al.* Voxelwise meta-analysis of gray matter anomalies in Alzheimer’s disease and mild cognitive impairment using anatomic likelihood estimation. J Neurol Sci 316, 21–29 (2012). 22385679 10.1016/j.jns.2012.02.010

[b8] Lerch J. P. *et al.* Focal decline of cortical thickness in Alzheimer’s disease identified by computational neuroanatomy. Cereb Cortex 15, 995–1001 (2005). 15537673 10.1093/cercor/bhh200

[b9] Ming J., Harms M. P., Morris J. C., Beg M. F. & Wang L. Integrated cortical structural marker for Alzheimer’s disease. Neurobiol Aging 36 Suppl 1, S53–59 (2015). 25444604 10.1016/j.neurobiolaging.2014.03.042 PMC4268172

[b10] Jack C. R. Jr. *et al.* Tracking pathophysiological processes in Alzheimer’s disease: an updated hypothetical model of dynamic biomarkers. Lancet Neurol 12, 207–216 (2013). 23332364 10.1016/S1474-4422(12)70291-0 PMC3622225

[b11] Biswal B., Yetkin F. Z., Haughton V. M. & Hyde J. S. Functional connectivity in the motor cortex of resting human brain using echo-planar MRI. Magn Reson Med 34, 537–541 (1995). 8524021 10.1002/mrm.1910340409

[b12] Fox M. D. & Raichle M. E. Spontaneous fluctuations in brain activity observed with functional magnetic resonance imaging. Nat Rev Neurosci 8, 700–711 (2007). 17704812 10.1038/nrn2201

[b13] Damoiseaux J. S. *et al.* Consistent resting-state networks across healthy subjects. Proc Natl Acad Sci USA 103, 13848–13853 (2006). 16945915 10.1073/pnas.0601417103 PMC1564249

[b14] Smith S. M. *et al.* Correspondence of the brain’s functional architecture during activation and rest. Proc Natl Acad Sci USA 106, 13040–13045 (2009). 19620724 10.1073/pnas.0905267106 PMC2722273

[b15] Menon V. Developmental pathways to functional brain networks: emerging principles. Trends Cogn Sci 17, 627–640 (2013). 24183779 10.1016/j.tics.2013.09.015

[b16] Menon V. Large-scale brain networks and psychopathology: a unifying triple network model. Trends Cogn Sci 15, 483–506 (2011). 21908230 10.1016/j.tics.2011.08.003

[b17] Barkhof F., Haller S. & Rombouts S. A. Resting-state functional MR imaging: a new window to the brain. Radiology 272, 29–49 (2014). 24956047 10.1148/radiol.14132388

[b18] Sorg C. *et al.* Selective changes of resting-state networks in individuals at risk for Alzheimer’s disease. Proc Natl Acad Sci USA 104, 18760–18765 (2007). 18003904 10.1073/pnas.0708803104 PMC2141850

[b19] Zhu D. C., Majumdar S., Korolev I. O., Berger K. L. & Bozoki A. C. Alzheimer’s disease and amnestic mild cognitive impairment weaken connections within the default-mode network: a multi-modal imaging study. J Alzheimers Dis 34, 969–984 (2013). 23313926 10.3233/JAD-121879

[b20] Greicius M. D., Srivastava G., Reiss A. L. & Menon V. Default-mode network activity distinguishes Alzheimer’s disease from healthy aging: evidence from functional MRI. Proc Natl Acad Sci USA 101, 4637–4642 (2004). 15070770 10.1073/pnas.0308627101 PMC384799

[b21] Rombouts S. A., Barkhof F., Goekoop R., Stam C. J. & Scheltens P. Altered resting state networks in mild cognitive impairment and mild Alzheimer’s disease: an fMRI study. Hum Brain Mapp 26, 231–239 (2005). 15954139 10.1002/hbm.20160 PMC6871685

[b22] Li R. *et al.* Attention-related networks in Alzheimer’s disease: a resting functional MRI study. Hum Brain Mapp 33, 1076–1088 (2012). 21538702 10.1002/hbm.21269 PMC3150638

[b23] Van Dam N. T. *et al.* Functional neural correlates of attentional deficits in amnestic mild cognitive impairment. PLoS One 8, e54035 (2013). 23326568 10.1371/journal.pone.0054035 PMC3543395

[b24] Weiler M. *et al.* Default mode, executive function, and language functional connectivity networks are compromised in mild Alzheimer’s disease. Curr Alzheimer Res 11, 274–282 (2014). 24484280 10.2174/1567205011666140131114716

[b25] He X. *et al.* Abnormal salience network in normal aging and in amnestic mild cognitive impairment and Alzheimer’s disease. Hum Brain Mapp 35, 3446–3464 (2014). 24222384 10.1002/hbm.22414 PMC6869630

[b26] Balthazar M. L. *et al.* Neuropsychiatric symptoms in Alzheimer’s disease are related to functional connectivity alterations in the salience network. Hum Brain Mapp 35, 1237–1246 (2014). 23418130 10.1002/hbm.22248 PMC6868965

[b27] Delbeuck X., Collette F. & Van der Linden M. Is Alzheimer’s disease a disconnection syndrome? Evidence from a crossmodal audio-visual illusory experiment. Neuropsychologia 45, 3315–3323 (2007). 17765932 10.1016/j.neuropsychologia.2007.05.001

[b28] Delbeuck X., Van der Linden M. & Collette F. Alzheimer’s disease as a disconnection syndrome. Neuropsychol Rev 13, 79–92 (2003). 12887040 10.1023/a:1023832305702

[b29] Wang K. *et al.* Altered functional connectivity in early Alzheimer’s disease: a resting-state fMRI study. Hum Brain Mapp 28, 967–978 (2007). 17133390 10.1002/hbm.20324 PMC6871392

[b30] Li R. *et al.* Alterations of directional connectivity among resting-state networks in Alzheimer disease. AJNR Am J Neuroradiol 34, 340–345 (2013). 22790250 10.3174/ajnr.A3197 PMC4097966

[b31] Dai Z. & He Y. Disrupted structural and functional brain connectomes in mild cognitive impairment and Alzheimer’s disease. Neurosci Bull 30, 217–232 (2014). 24733652 10.1007/s12264-013-1421-0 PMC5562665

[b32] He Y., Chen Z. & Evans A. Structural insights into aberrant topological patterns of large-scale cortical networks in Alzheimer’s disease. J Neurosci 28, 4756–4766 (2008). 18448652 10.1523/JNEUROSCI.0141-08.2008 PMC6670444

[b33] Wang J. *et al.* Apolipoprotein E epsilon4 modulates functional brain connectome in Alzheimer’s disease. Hum Brain Mapp 36, 1828–1846 (2015). 25619771 10.1002/hbm.22740 PMC6869368

[b34] Liu Y. *et al.* Impaired long distance functional connectivity and weighted network architecture in Alzheimer’s disease. Cereb Cortex 24, 1422–1435 (2014). 23314940 10.1093/cercor/bhs410 PMC4215108

[b35] Zhao X. *et al.* Disrupted small-world brain networks in moderate Alzheimer’s disease: a resting-state FMRI study. PLoS One 7, e33540 (2012). 22457774 10.1371/journal.pone.0033540 PMC3311642

[b36] Brier M. R. *et al.* Loss of intranetwork and internetwork resting state functional connections with Alzheimer’s disease progression. J Neurosci 32, 8890–8899 (2012). 22745490 10.1523/JNEUROSCI.5698-11.2012 PMC3458508

[b37] Thomas J. B. *et al.* Functional connectivity in autosomal dominant and late-onset Alzheimer disease. JAMA Neurol 71, 1111–1122 (2014). 25069482 10.1001/jamaneurol.2014.1654 PMC4240274

[b38] Brier M. R. *et al.* Unrecognized preclinical Alzheimer disease confounds rs-fcMRI studies of normal aging. Neurology 83, 1613–1619 (2014). 25261500 10.1212/WNL.0000000000000939 PMC4223085

[b39] Wang L. *et al.* Alzheimer disease family history impacts resting state functional connectivity. Ann Neurol 72, 571–577 (2012). 23109152 10.1002/ana.23643 PMC3490438

[b40] Lim H. K. *et al.* Regional amyloid burden and intrinsic connectivity networks in cognitively normal elderly subjects. Brain 137, 3327–3338 (2014). 25266592 10.1093/brain/awu271 PMC4240287

[b41] Elman J. A. *et al.* Effects of Beta-Amyloid on Resting State Functional Connectivity Within and Between Networks Reflect Known Patterns of Regional Vulnerability. Cereb Cortex (2014). 10.1093/cercor/bhu259 PMC4712800 25405944

[b42] Drzezga A. *et al.* Neuronal dysfunction and disconnection of cortical hubs in non-demented subjects with elevated amyloid burden. Brain 134, 1635–1646 (2011). 21490054 10.1093/brain/awr066 PMC3102239

[b43] Wang P. *et al.* Perceptual and response interference in Alzheimer’s disease and mild cognitive impairment. Clin Neurophysiol 124, 2389–2396 (2013). 23786793 10.1016/j.clinph.2013.05.014

[b44] Zhang Z. *et al.* Altered spontaneous activity in Alzheimer’s disease and mild cognitive impairment revealed by Regional Homogeneity. Neuroimage 59, 1429–1440 (2012). 21907292 10.1016/j.neuroimage.2011.08.049

[b45] Yao H. *et al.* Decreased functional connectivity of the amygdala in Alzheimer’s disease revealed by resting-state fMRI. Eur J Radiol 82, 1531–1538 (2013). 23643516 10.1016/j.ejrad.2013.03.019

[b46] Zhou B. *et al.* Impaired functional connectivity of the thalamus in alzheimer’ s disease and mild cognitive impairment: a resting-state FMRI study. Curr Alzheimer Res 10, 754–766 (2013). 23905993 10.2174/15672050113109990146

[b47] Zhang Z. *et al.* Altered functional connectivity of the marginal division in Alzheimer’s disease. Curr Alzheimer Res 11, 145–155 (2014). 24410630 10.2174/1567205011666140110112608

[b48] Guo Y. *et al.* Grey-matter volume as a potential feature for the classification of Alzheimer’s disease and mild cognitive impairment: an exploratory study. Neurosci Bull 30, 477–489 (2014). 24760581 10.1007/s12264-013-1432-x PMC5562611

[b49] Albert M. S. *et al.* The diagnosis of mild cognitive impairment due to Alzheimer’s disease: recommendations from the National Institute on Aging-Alzheimer’s Association workgroups on diagnostic guidelines for Alzheimer’s disease. Alzheimers Dement 7, 270–279 (2011). 21514249 10.1016/j.jalz.2011.03.008 PMC3312027

[b50] McKhann G. M. *et al.* The diagnosis of dementia due to Alzheimer’s disease: recommendations from the National Institute on Aging-Alzheimer’s Association workgroups on diagnostic guidelines for Alzheimer’s disease. Alzheimers Dement 7, 263–269 (2011). 21514250 10.1016/j.jalz.2011.03.005 PMC3312024

[b51] Van Dijk K. R., Sabuncu M. R. & Buckner R. L. The influence of head motion on intrinsic functional connectivity MRI. Neuroimage 59, 431–438 (2012). 21810475 10.1016/j.neuroimage.2011.07.044 PMC3683830

[b52] Wang L. *et al.* The effect of APOE epsilon4 allele on cholinesterase inhibitors in patients with Alzheimer disease: evaluation of the feasibility of resting state functional connectivity magnetic resonance imaging. Alzheimer Dis Assoc Disord 28, 122–127 (2014). 24830360 10.1097/WAD.0b013e318299d096 PMC4024181

[b53] Buckner R. L., Andrews-Hanna J. R. & Schacter D. L. The brain’s default network: anatomy, function, and relevance to disease. Ann N Y Acad Sci 1124, 1–38 (2008). 18400922 10.1196/annals.1440.011

[b54] Sestieri C., Corbetta M., Romani G. L. & Shulman G. L. Episodic memory retrieval, parietal cortex, and the default mode network: functional and topographic analyses. J Neurosci 31, 4407–4420 (2011). 21430142 10.1523/JNEUROSCI.3335-10.2011 PMC3098040

[b55] Buckner R. L. *et al.* Molecular, structural, and functional characterization of Alzheimer’s disease: evidence for a relationship between default activity, amyloid, and memory. J Neurosci 25, 7709–7717 (2005). 16120771 10.1523/JNEUROSCI.2177-05.2005 PMC6725245

[b56] Mormino E. C. *et al.* Relationships between beta-amyloid and functional connectivity in different components of the default mode network in aging. Cereb Cortex 21, 2399–2407 (2011). 21383234 10.1093/cercor/bhr025 PMC3169663

[b57] Sperling R. A. *et al.* Amyloid deposition is associated with impaired default network function in older persons without dementia. Neuron 63, 178–188 (2009). 19640477 10.1016/j.neuron.2009.07.003 PMC2738994

[b58] Sheline Y. I. *et al.* Amyloid plaques disrupt resting state default mode network connectivity in cognitively normal elderly. Biol Psychiatry 67, 584–587 (2010). 19833321 10.1016/j.biopsych.2009.08.024 PMC2829379

[b59] Koechlin E. & Summerfield C. An information theoretical approach to prefrontal executive function. Trends Cogn Sci 11, 229–235 (2007). 17475536 10.1016/j.tics.2007.04.005

[b60] Miller E. K. & Cohen J. D. An integrative theory of prefrontal cortex function. Annu Rev Neurosci 24, 167–202 (2001). 11283309 10.1146/annurev.neuro.24.1.167

[b61] Bunge S. A., Ochsner K. N., Desmond J. E., Glover G. H. & Gabrieli J. D. Prefrontal regions involved in keeping information in and out of mind. Brain 124, 2074–2086 (2001). 11571223 10.1093/brain/124.10.2074

[b62] Morris J. C. Mild cognitive impairment is early-stage Alzheimer disease: time to revise diagnostic criteria. Arch Neurol 63, 15–16 (2006). 16401731 10.1001/archneur.63.1.15

[b63] Pariente J. *et al.* Alzheimer’s patients engage an alternative network during a memory task. Ann Neurol 58, 870–879 (2005). 16315273 10.1002/ana.20653

[b64] Woodard J. L. *et al.* Compensatory recruitment of neural resources during overt rehearsal of word lists in Alzheimer’s disease. Neuropsychology 12, 491–504 (1998). 9805319 10.1037//0894-4105.12.4.491

[b65] Marshall G. A. *et al.* Executive function and instrumental activities of daily living in mild cognitive impairment and Alzheimer’s disease. Alzheimers Dement 7, 300–308 (2011). 21575871 10.1016/j.jalz.2010.04.005 PMC3096844

[b66] Perry R. J. & Hodges J. R. Attention and executive deficits in Alzheimer’s disease. A critical review. Brain 122 (Pt 3), 383–404 (1999). 10094249 10.1093/brain/122.3.383

[b67] Li S. J. *et al.* Alzheimer Disease: evaluation of a functional MR imaging index as a marker. Radiology 225, 253–259 (2002). 12355013 10.1148/radiol.2251011301

[b68] Devanand D. P. *et al.* Combining early markers strongly predicts conversion from mild cognitive impairment to Alzheimer’s disease. Biol Psychiatry 64, 871–879 (2008). 18723162 10.1016/j.biopsych.2008.06.020 PMC2613777

[b69] Lin F. R. *et al.* Hearing loss and incident dementia. Arch Neurol 68, 214–220 (2011). 21320988 10.1001/archneurol.2010.362 PMC3277836

[b70] Verghese J., Wang C., Lipton R. B., Holtzer R. & Xue X. Quantitative gait dysfunction and risk of cognitive decline and dementia. J Neurol Neurosurg Psychiatry 78, 929–935 (2007). 17237140 10.1136/jnnp.2006.106914 PMC1995159

[b71] Li W., Howard J. D. & Gottfried J. A. Disruption of odour quality coding in piriform cortex mediates olfactory deficits in Alzheimer’s disease. Brain 133, 2714–2726 (2010). 20724290 10.1093/brain/awq209 PMC2948816

[b72] Albers M. W. *et al.* At the interface of sensory and motor dysfunctions and Alzheimer’s disease. Alzheimers Dement 11, 70–98 (2015). 25022540 10.1016/j.jalz.2014.04.514 PMC4287457

[b73] Zhou J. & Seeley W. W. Network dysfunction in Alzheimer’s disease and frontotemporal dementia: implications for psychiatry. Biol Psychiatry 75, 565–573 (2014). 24629669 10.1016/j.biopsych.2014.01.020

[b74] Cabeza R., Ciaramelli E., Olson I. R. & Moscovitch M. The parietal cortex and episodic memory: an attentional account. Nat Rev Neurosci 9, 613–625 (2008). 18641668 10.1038/nrn2459 PMC2692883

[b75] Baddeley A. D., Baddeley H. A., Bucks R. S. & Wilcock G. K. Attentional control in Alzheimer’s disease. Brain 124, 1492–1508 (2001). 11459742 10.1093/brain/124.8.1492

[b76] Galton C. J., Patterson K., Xuereb J. H. & Hodges J. R. Atypical and typical presentations of Alzheimer’s disease: a clinical, neuropsychological, neuroimaging and pathological study of 13 cases. Brain 123 Pt 3, 484–498 (2000). 10686172 10.1093/brain/123.3.484

[b77] Agosta F. *et al.* Resting state fMRI in Alzheimer’s disease: beyond the default mode network. Neurobiol Aging 33, 1564–1578 (2012). 21813210 10.1016/j.neurobiolaging.2011.06.007

[b78] Myers N. *et al.* Within-patient correspondence of amyloid-beta and intrinsic network connectivity in Alzheimer’s disease. Brain 137, 2052–2064 (2014). 24771519 10.1093/brain/awu103 PMC4065018

[b79] Hedden T. *et al.* Disruption of functional connectivity in clinically normal older adults harboring amyloid burden. J Neurosci 29, 12686–12694 (2009). 19812343 10.1523/JNEUROSCI.3189-09.2009 PMC2808119

[b80] Menon V. & Uddin L. Q. Saliency, switching, attention and control: a network model of insula function. Brain Struct Funct 214, 655–667 (2010). 20512370 10.1007/s00429-010-0262-0 PMC2899886

[b81] Uddin L. Q. Salience processing and insular cortical function and dysfunction. Nat Rev Neurosci 16, 55–61 (2015). 25406711 10.1038/nrn3857

[b82] Bonnelle V. *et al.* Salience network integrity predicts default mode network function after traumatic brain injury. Proc Natl Acad Sci USA 109, 4690–4695 (2012). 22393019 10.1073/pnas.1113455109 PMC3311356

[b83] Seeley W. W. *et al.* Dissociable intrinsic connectivity networks for salience processing and executive control. J Neurosci 27, 2349–2356 (2007). 17329432 10.1523/JNEUROSCI.5587-06.2007 PMC2680293

[b84] Liang X., Zou Q., He Y. & Yang Y. Topologically Reorganized Connectivity Architecture of Default-Mode, Executive-Control, and Salience Networks across Working Memory Task Loads. Cereb Cortex (2015). 10.1093/cercor/bhu316 PMC4785946 25596593

[b85] Sridharan D., Levitin D. J. & Menon V. A critical role for the right fronto-insular cortex in switching between central-executive and default-mode networks. Proc Natl Acad Sci USA 105, 12569–12574 (2008). 18723676 10.1073/pnas.0800005105 PMC2527952

[b86] Lehmann M. *et al.* Intrinsic connectivity networks in healthy subjects explain clinical variability in Alzheimer’s disease. Proc Natl Acad Sci USA 110, 11606–11611 (2013). 23798398 10.1073/pnas.1221536110 PMC3710820

[b87] Brier M. R. *et al.* Functional connectivity and graph theory in preclinical Alzheimer’s disease. Neurobiol Aging 35, 757–768 (2014). 24216223 10.1016/j.neurobiolaging.2013.10.081 PMC3880636

[b88] Tombaugh T. N. & McIntyre N. J. The mini-mental state examination: a comprehensive review. J Am Geriatr Soc 40, 922–935 (1992). 1512391 10.1111/j.1532-5415.1992.tb01992.x

[b89] Song J. *et al.* Aberrant functional organization within and between resting-state networks in AD. PLoS One 8, e63727 (2013). 23667665 10.1371/journal.pone.0063727 PMC3647055

[b90] Trachtenberg A. J., Filippini N. & Mackay C. E. The effects of APOE-epsilon4 on the BOLD response. Neurobiol Aging 33, 323–334 (2012). 20409610 10.1016/j.neurobiolaging.2010.03.009

[b91] Sheline Y. I. *et al.* APOE4 allele disrupts resting state fMRI connectivity in the absence of amyloid plaques or decreased CSF Abeta42. J Neurosci 30, 17035–17040 (2010). 21159973 10.1523/JNEUROSCI.3987-10.2010 PMC3023180

