# Supplementary material for: Aberrant intra- and inter-network connectivity architectures in Alzheimer’s disease and mild cognitive impairment
Source: Sci Rep. 2015 Oct 6;5:14824. doi: 10.1038/srep14824 (PMC4594099; doi:10.1038/srep14824)
Supplement: Supplementary Information [file srep14824-s1.doc]

**Aberrant intra- and inter-network connectivity architectures in Alzheimer’s disease and mild cognitive impairment**

Pan Wang^1,2*^, Bo Zhou^1*^, Hongxiang Yao^3^, Yafeng Zhan^4,5^, Zengqiang Zhang^1,6^, Yue Cui^4,7^, Kaibin Xu^4,7^, Jianhua Ma^5^, Luning Wang^1^, Ningyu An^3^, Xi Zhang^1, CA^, Yong Liu^4, 7, CA^, Tianzi Jiang^4, 7, 8^

^1^Department of Neurology, Institute of Geriatrics and Gerontology, Chinese PLA General Hospital, Beijing, 100853, China

^2^Department of Neurology, Tianjin Huanhu Hospital, Tianjin, 300060, China

^3^Department of Radiology, Chinese PLA General Hospital, Beijing, 100853, China

^4^Brainnetome Center, Institute of Automation, Chinese Academy of Sciences, Beijing, 100190, China

^5^School of Biomedical Engineering, Southern Medical University, Guangzhou, Guangdong, 510515, China

^6^Hainan Branch of Chinese PLA General Hospital, Sanya, 572014, China

^7^National Laboratory of Pattern Recognition, Institute of Automation, Chinese Academy of Sciences, Beijing, 100190, China

^8^CAS Center for Excellence in Brain Science, Institute of Automation, Chinese Academy of Sciences, Beijing 100190, China.

* These authors contributed equally to this work.

**Correspondence to**

Dr. Xi Zhang

Department of Neurology, Institute of Geriatrics and Gerontology, Chinese PLA General Hospital, Beijing, 100853, China

E-mail: [zhangxi@301hospital.com.cn](mailto:zhangxi@301hospital.com.cn)

Dr. Yong Liu

Brainnetome Center, Institute of Automation, Chinese Academy of Sciences, Beijing, 100190, China

Email: [yliu@nlpr.ia.ac.cn](mailto:yliu@nlpr.ia.ac.cn)

Outline

[**Aberrant intra- and inter-network connectivity architectures in Alzheimer’s disease and mild cognitive impairment** 1](#__RefHeading___Toc424914276)

[Detail of dataset 3](#__RefHeading___Toc424914277)

[Table S1. 5](#__RefHeading___Toc424914278)

[Table S2 6](#__RefHeading___Toc424914279)

[Figure S1. 9](#__RefHeading___Toc424914280)

[Figure S2 10](#__RefHeading___Toc424914281)

[Reference 11](#__RefHeading___Toc424914282)

# Detail of dataset

This study was approved by the Medical Ethics Committee of the Chinese PLA General Hospital. All participants were assessed in Chinese PLA General Hospital, and written consent forms were obtained from the subjects or their legal guardians (the wife/husband or children). A subset of the subjects were included in our previous studies on perceptual and response interference in AD [^1^](#_ENREF_1), changes in regional brain activity [^2^](#_ENREF_2) and the functional connectivity patterns of the amygdala [^3^](#_ENREF_3); moreover, the same dataset has been used to study the functional connectivity patterns of the thalamus [^4^](#_ENREF_4), and marginal division [^5^](#_ENREF_5) in the resting state. Here, we provide a detailed introduction regarding data inclusion/exclusion criteria, acquisition and processing; this information was also provided in our previous studies.

We recruited the subjects from two sources: outpatients from the Chinese PLA General Hospital or recruitment through a website advertisement (http://www.301ad.com.cn, Chinese version). Before they were selected for this study, all participants underwent general physical, psychological and laboratory examinations. All subjects were right handed and underwent a neuropsychological test battery that included the Mini-Mental State Examination (MMSE), Auditory Verbal Learning Test (AVLT), Geriatric Depression Scale (GDS), Clinical Dementia Rating (CDR) [^6^](#_ENREF_6) and Activities of Daily Living scale (ADL).

All subjects met identical methodological stringency criteria and met the core clinical criteria for probable AD dementia and MCI as described in the recently published recommendation of the new diagnostic criteria [^7-9^](#_ENREF_7). The recruited AD patients fulfilled the following inclusion criteria: (1) were diagnosed using the National Institute of Neurological and Communicative Disorders and Stroke and the Alzheimer’s Disease and Related Disorders Association criteria for probable AD; (2) had CDR = 1 or CDR = 2; (3) were not receiving nootropic drugs such as anticholinesterase inhibitors; and (4) were able to perform the neuropsychological test. The MCI diagnostic criteria that were used in this study were described by Petersen et al. [^10^](#_ENREF_10) and included the following components: (1) memory complaints lasting at least 6 months, (2) CDR = 0.5, (3) intact functional status and ADL < 26 and (4) the absence of dementia. The NC criteria were as follows: (1) normal general physical status, (2) CDR = 0 and (3) no memory complaints. Simultaneously, all subjects were evaluated by two senior neurologists and were not treated with any medication that might influence their cognition during the task.

The exclusion criteria for this study were as follows: (1) metabolic conditions such as hypothyroidism or vitamin B12 or folic acid deficiencies; (2) psychiatric disorders such as schizophrenia or depression; (3) infarction or brain hemorrhaging as indicated by MR/CT imaging; or (4) Parkinsonian syndrome, epilepsy or other nervous system diseases that could influence cognitive function. In addition, patients with a metallic foreign body, such as a cochlear implant, heart stent or other MR scanning-relevant contraindications were excluded from the study.

After excluding subjects with large head motion, 89 subjects, including 35 AD patients, 27 MCI subjects, and 27 age- and gender-matched NC subjects, were included for further analysis.

# Table S1.

Table S1 Regions and MNI coordinates of ROIs (see **Brier et al.,** [**^11^**](#_ENREF_11) )

| **ROI** | **MNI coordinates** |
| --- | --- |
| Posterior cingulate cortex^a^ | 0, -51, 29 |
| Medial prefrontal cortex (mPFC)^a^ | 0, 61, 22 |
| Left lateral parietal (lLP)^a^ | 48,66, 34 |
| Right lateral parietal (rLP)^a^ | 53,61, 35 |
| Left inferior temporal (liTmp)^a^ | 65,22,9 |
| Right inferior temporal (riTmp)^a^ | 61,21,12 |
| Medial thalamus (mdThal)^a^ | 0,9, 7 |
| Left posterior cerebellum (lpCBLM)^a^ | 28,82,32 |
| Right posterior cerebellum (rpCBLM)^a^ | 26,89,34 |
| Left front eye field (lFEF)^b^ | 29,5, 55 |
| Right front eye field (rFEF)^b^ | 31,5, 54 |
| Left posterior intraparietal sulcus (lpIPS)^b^ | 26,65, 52 |
| Right posterior intraparietal sulcus (rpIPS)^b^ | 28,65, 51 |
| Left anterior intraparietal sulcus (laIPS)^b^ | 45,37, 48 |
| Right anterior intraparietal sulcus (raIPS)^b^ | 43,36, 46 |
| Left MT (lMT)^b^ | 52,66,4 |
| Right MT (rMT)^b^ | 55,62,7 |
| Dorsal mPFC (dmPFC)^c^ | 1, 30, 44 |
| Left anterior PFC (laPFC)^c^ | 45, 50,5 |
| Right anterior PFC (raPFC)^c^ | 46, 51,7 |
| Left superior parietal (lSP)^c^ | 51,50, 49 |
| Right superior Parietal (rSP)^c^ | 53,49, 47 |
| Right anterior cingulate cortex (rPG-ACC)^d^ | 12, 32, 30 |
| Left anterior cingulate cortex (LPG-ACC)^d^ | 13, 34, 16 |
| Right ventral anterior cingulate cortex (rSG-ACC)^d^ | 10, 34,6 |
| Left putamen (lPut)^d^ | 19, 3, 9 |
| Right putamen (rPut)^d^ | 25, 18, 8 |
| Left insula (lIns)^d^ | 42, 6, 4 |
| Right insula (rIns)^d^ | 43, 7, 2 |
| Left motor cortex (lMC)^e^ | 40,23, 53 |
| Right motor cortex (rMC)^e^ | 41,22, 48 |
| Supplemental motor area (SMA)^e^ | 1,18, 49 |
| Left primary visual (lV1)^e^ | 8,83, 0 |
| Right primary visual (rV1)^e^ | 7,83, 0 |
| Left primary auditory (lA1)^e^ | 64,28, 13 |
| Right primary auditory (rA1)^e^ | 62,24, 13 |

a: default mode network (DMN); b: dorsal attention network (DAN); c: control network (CON); d: salience network (SAL); and e: sensorimotor network (SMN).

# Table S2

Table S2, The impaired connectivity AD, also the correlation between MMSE and strength of the functional connectivity.

| Node_1 | Node_2 | P | P_randperm | NC_mean | NC_sd | MCI  mean | MCI  sd | AD  mean | AD  sd | MMSE  MC_ZR | MMSE  MC_P | MMSE  AD_ZR | MMSE  AD_P | MMSE  MC_AD_ZR | MMSE  MC_AD_P | Type |
| --- | --- | --- | --- | --- | --- | --- | --- | --- | --- | --- | --- | --- | --- | --- | --- | --- |
| dmPFC | SMA | 0.001 | 0.001 | 1.086 | 0.270 | 1.094 | 0.214 | 1.300 | 0.268 | -0.036 | 0.857 | -0.037 | 0.834 | -0.304 | 0.016 | CON-SMN |
| rpIPS | rMC | 0.001 | 0.001 | 1.449 | 0.285 | 1.211 | 0.219 | 1.262 | 0.236 | 0.292 | 0.140 | 0.040 | 0.818 | -0.017 | 0.897 | DAN-SMN |
| liTmp | rpIPS | 0.002 | 0.002 | 0.491 | 0.228 | 0.279 | 0.192 | 0.322 | 0.249 | -0.110 | 0.584 | -0.153 | 0.379 | -0.167 | 0.196 | DMN-DAN |
| SMA | lV1 | 0.005 | 0.004 | 0.508 | 0.271 | 0.415 | 0.218 | 0.300 | 0.236 | 0.041 | 0.841 | 0.144 | 0.408 | 0.255 | 0.046 | SMN-SMN |
| rPut | rIns | 0.005 | 0.006 | 2.352 | 0.227 | 2.137 | 0.248 | 2.185 | 0.267 | 0.238 | 0.232 | -0.173 | 0.321 | -0.117 | 0.367 | SAL-SAL |
| PCC | rPut | 0.006 | 0.006 | 0.414 | 0.182 | 0.476 | 0.226 | 0.603 | 0.265 | 0.110 | 0.585 | 0.338 | 0.047 | 0.002 | 0.987 | DMN-SAL |
| lMC | rMC | 0.006 | 0.006 | 2.715 | 0.291 | 2.495 | 0.299 | 2.515 | 0.245 | -0.081 | 0.689 | -0.222 | 0.200 | -0.139 | 0.280 | SMN-SMN |
| lpCBLM | raPFC | 0.006 | 0.005 | 0.474 | 0.227 | 0.430 | 0.266 | 0.284 | 0.233 | 0.144 | 0.475 | -0.043 | 0.808 | 0.213 | 0.097 | DMN-CON |
| riTmp | lACC | 0.007 | 0.006 | 0.265 | 0.256 | 0.303 | 0.236 | 0.120 | 0.225 | 0.199 | 0.321 | 0.211 | 0.224 | 0.394 | 0.002 | DMN-SAL |
| rpIPS | SMA | 0.008 | 0.007 | 1.736 | 0.256 | 1.541 | 0.184 | 1.537 | 0.322 | 0.220 | 0.271 | -0.032 | 0.855 | 0.011 | 0.931 | DAN-SMN |
| laIPS | rMT | 0.008 | 0.007 | -0.823 | 0.253 | -1.041 | 0.245 | -0.983 | 0.280 | 0.190 | 0.343 | -0.140 | 0.421 | -0.122 | 0.343 | DAN-DAN |
| laIPS | rMC | 0.009 | 0.009 | 2.238 | 0.226 | 2.045 | 0.356 | 2.028 | 0.248 | -0.079 | 0.695 | -0.233 | 0.178 | -0.088 | 0.497 | DAN-SMN |
| mPFC | rPut | 0.009 | 0.009 | 0.585 | 0.228 | 0.647 | 0.163 | 0.736 | 0.179 | 0.100 | 0.618 | -0.171 | 0.327 | -0.251 | 0.049 | DMN-SAL |
| lpCBLM | lSP | 0.010 | 0.009 | -0.429 | 0.275 | -0.569 | 0.241 | -0.631 | 0.253 | -0.021 | 0.917 | -0.225 | 0.193 | -0.022 | 0.864 | DMN-CON |
| PCC | lV1 | 0.012 | 0.010 | 0.127 | 0.247 | -0.065 | 0.221 | 0.014 | 0.228 | 0.168 | 0.403 | 0.015 | 0.930 | -0.094 | 0.469 | DMN-SMN |
| laPFC | lSP | 0.012 | 0.013 | 0.865 | 0.257 | 1.102 | 0.314 | 0.948 | 0.299 | 0.240 | 0.228 | 0.072 | 0.680 | 0.251 | 0.049 | CON-CON |
| SMA | rV1 | 0.013 | 0.012 | 0.324 | 0.261 | 0.217 | 0.263 | 0.118 | 0.273 | 0.215 | 0.282 | 0.090 | 0.606 | 0.209 | 0.103 | SMN-SMN |
| PCC | rV1 | 0.014 | 0.017 | -0.018 | 0.228 | -0.210 | 0.226 | -0.129 | 0.251 | 0.177 | 0.378 | 0.132 | 0.450 | -0.030 | 0.820 | DMN-SMN |
| liTmp | laPFC | 0.016 | 0.017 | 1.790 | 0.190 | 1.585 | 0.299 | 1.607 | 0.332 | 0.022 | 0.913 | 0.040 | 0.819 | -0.002 | 0.989 | DMN-CON |
| PCC | riTmp | 0.019 | 0.019 | 0.811 | 0.325 | 0.766 | 0.378 | 0.596 | 0.239 | 0.331 | 0.091 | 0.135 | 0.440 | 0.307 | 0.015 | DMN-DMN |
| SMA | rA1 | 0.020 | 0.019 | 0.797 | 0.336 | 0.585 | 0.290 | 0.614 | 0.273 | 0.245 | 0.218 | 0.286 | 0.096 | 0.137 | 0.288 | SMN-SMN |
| rMT | rV1 | 0.020 | 0.022 | -0.161 | 0.279 | -0.357 | 0.242 | -0.220 | 0.257 | 0.150 | 0.456 | -0.280 | 0.103 | -0.305 | 0.016 | DAN-SMN |
| lpIPS | lV1 | 0.020 | 0.020 | 0.927 | 0.284 | 0.741 | 0.238 | 0.774 | 0.250 | -0.191 | 0.341 | -0.123 | 0.481 | -0.141 | 0.275 | DAN-SMN |
| lpIPS | rMC | 0.021 | 0.020 | 2.013 | 0.241 | 1.827 | 0.238 | 1.887 | 0.258 | -0.032 | 0.875 | 0.051 | 0.770 | -0.067 | 0.606 | DAN-SMN |
| laPFC | rSP | 0.021 | 0.018 | 0.940 | 0.215 | 1.108 | 0.278 | 0.979 | 0.194 | 0.277 | 0.162 | 0.066 | 0.708 | 0.270 | 0.034 | CON-CON |
| liTmp | rV1 | 0.024 | 0.025 | 1.415 | 0.268 | 1.242 | 0.233 | 1.266 | 0.249 | -0.286 | 0.148 | 0.172 | 0.322 | 0.005 | 0.970 | DMN-SMN |
| riTmp | laPFC | 0.024 | 0.023 | 0.496 | 0.231 | 0.396 | 0.265 | 0.322 | 0.235 | 0.259 | 0.191 | 0.084 | 0.631 | 0.190 | 0.138 | DMN-CON |
| rpCBLM | rSP | 0.025 | 0.028 | -1.140 | 0.288 | -1.045 | 0.196 | -0.968 | 0.236 | -0.297 | 0.133 | -0.224 | 0.195 | -0.284 | 0.025 | DMN-CON |
| laIPS | lV1 | 0.026 | 0.024 | 0.763 | 0.294 | 0.588 | 0.217 | 0.616 | 0.245 | -0.200 | 0.317 | 0.080 | 0.646 | -0.033 | 0.797 | DAN-SMN |
| rpCBLM | laIPS | 0.028 | 0.030 | -0.050 | 0.264 | -0.095 | 0.225 | 0.076 | 0.277 | -0.055 | 0.786 | 0.042 | 0.809 | -0.219 | 0.087 | DMN-DAN |
| lpIPS | rV1 | 0.028 | 0.026 | 0.547 | 0.259 | 0.359 | 0.281 | 0.439 | 0.227 | -0.308 | 0.118 | -0.062 | 0.725 | -0.196 | 0.128 | DAN-SMN |
| lSP | rSP | 0.028 | 0.028 | 1.870 | 0.276 | 1.907 | 0.289 | 1.732 | 0.248 | 0.473 | 0.013 | -0.132 | 0.448 | 0.250 | 0.050 | CON-CON |
| rpIPS | lMC | 0.029 | 0.028 | 1.336 | 0.281 | 1.190 | 0.254 | 1.161 | 0.253 | 0.321 | 0.103 | 0.059 | 0.736 | 0.122 | 0.346 | DAN-SMN |
| rpCBLM | lMC | 0.029 | 0.032 | -0.207 | 0.258 | -0.200 | 0.208 | -0.061 | 0.254 | 0.137 | 0.495 | 0.063 | 0.718 | -0.157 | 0.223 | DMN-SMN |
| lFEF | laPFC | 0.032 | 0.032 | -0.419 | 0.184 | -0.371 | 0.266 | -0.516 | 0.202 | -0.153 | 0.445 | 0.252 | 0.144 | 0.293 | 0.021 | DAN-CON |
| mPFC | rSP | 0.033 | 0.033 | 0.442 | 0.310 | 0.648 | 0.277 | 0.568 | 0.276 | 0.147 | 0.465 | 0.022 | 0.900 | 0.138 | 0.286 | DMN-CON |
| lLP | rIns | 0.033 | 0.032 | -0.437 | 0.235 | -0.319 | 0.229 | -0.286 | 0.222 | 0.383 | 0.048 | -0.083 | 0.635 | -0.031 | 0.808 | DMN-SAL |
| mdThal | SMA | 0.035 | 0.038 | -0.769 | 0.256 | -0.728 | 0.251 | -0.597 | 0.296 | -0.113 | 0.576 | -0.129 | 0.460 | -0.249 | 0.051 | DMN-SMN |
| rPut | lA1 | 0.036 | 0.039 | 1.340 | 0.265 | 1.139 | 0.267 | 1.259 | 0.307 | 0.431 | 0.025 | 0.095 | 0.587 | -0.038 | 0.771 | SAL-SMN |
| lFEF | rV1 | 0.036 | 0.034 | 0.538 | 0.196 | 0.438 | 0.230 | 0.382 | 0.258 | 0.285 | 0.150 | 0.178 | 0.305 | 0.214 | 0.095 | DAN-SMN |
| lMC | lV1 | 0.037 | 0.038 | 1.584 | 0.295 | 1.485 | 0.190 | 1.409 | 0.283 | 0.162 | 0.421 | 0.009 | 0.961 | 0.137 | 0.289 | SMN-SMN |
| dmPFC | lSP | 0.038 | 0.041 | 0.747 | 0.355 | 0.757 | 0.306 | 0.578 | 0.266 | 0.043 | 0.832 | 0.155 | 0.374 | 0.294 | 0.021 | CON-CON |
| raPFC | rPut | 0.038 | 0.036 | 0.168 | 0.177 | 0.205 | 0.249 | 0.305 | 0.217 | 0.275 | 0.165 | 0.164 | 0.347 | -0.036 | 0.783 | CON-SAL |
| rMC | lV1 | 0.039 | 0.036 | 1.825 | 0.256 | 1.768 | 0.228 | 1.670 | 0.233 | 0.068 | 0.736 | 0.248 | 0.152 | 0.280 | 0.027 | SMN-SMN |
| rpIPS | laIPS | 0.043 | 0.043 | 0.999 | 0.362 | 0.782 | 0.274 | 0.868 | 0.307 | 0.212 | 0.288 | 0.345 | 0.042 | 0.098 | 0.447 | DAN-DAN |
| lpCBLM | lpIPS | 0.044 | 0.044 | 0.217 | 0.276 | 0.107 | 0.233 | 0.286 | 0.304 | 0.084 | 0.678 | 0.124 | 0.479 | -0.153 | 0.236 | DMN-DAN |
| liTmp | lpIPS | 0.045 | 0.047 | 1.023 | 0.215 | 0.859 | 0.235 | 0.891 | 0.294 | -0.072 | 0.722 | -0.165 | 0.342 | -0.140 | 0.277 | DMN-DAN |
| rpCBLM | rMT | 0.045 | 0.046 | 1.030 | 0.323 | 1.022 | 0.305 | 1.192 | 0.281 | 0.177 | 0.377 | -0.033 | 0.851 | -0.190 | 0.138 | DMN-DAN |
| mPFC | lpCBLM | 0.047 | 0.045 | -1.170 | 0.277 | -1.020 | 0.287 | -1.209 | 0.331 | -0.152 | 0.449 | 0.089 | 0.609 | 0.234 | 0.067 | DMN-DMN |
| rFEF | rMC | 0.047 | 0.046 | 3.000 | 0.309 | 2.827 | 0.274 | 2.972 | 0.248 | 0.023 | 0.911 | -0.092 | 0.600 | -0.235 | 0.066 | DAN-SMN |
| mPFC | rLP | 0.047 | 0.047 | -1.750 | 0.367 | -1.524 | 0.305 | -1.620 | 0.323 | 0.085 | 0.674 | 0.067 | 0.703 | 0.155 | 0.228 | DMN-DMN |
| lMC | rV1 | 0.048 | 0.048 | 1.387 | 0.292 | 1.275 | 0.263 | 1.198 | 0.319 | 0.052 | 0.799 | 0.051 | 0.770 | 0.128 | 0.321 | SMN-SMN |
| rpCBLM | rACC | 0.050 | 0.049 | -1.355 | 0.205 | -1.495 | 0.187 | -1.410 | 0.226 | -0.071 | 0.724 | -0.033 | 0.852 | -0.173 | 0.180 | DMN-SAL |

Note: The abbreviations are listed in table S1.

Orange color background mean the strength of the impaired functional connectivity significant correlated with MMSE.

The read font means that the simulated p value smaller than the real p value.

# Figure S1.


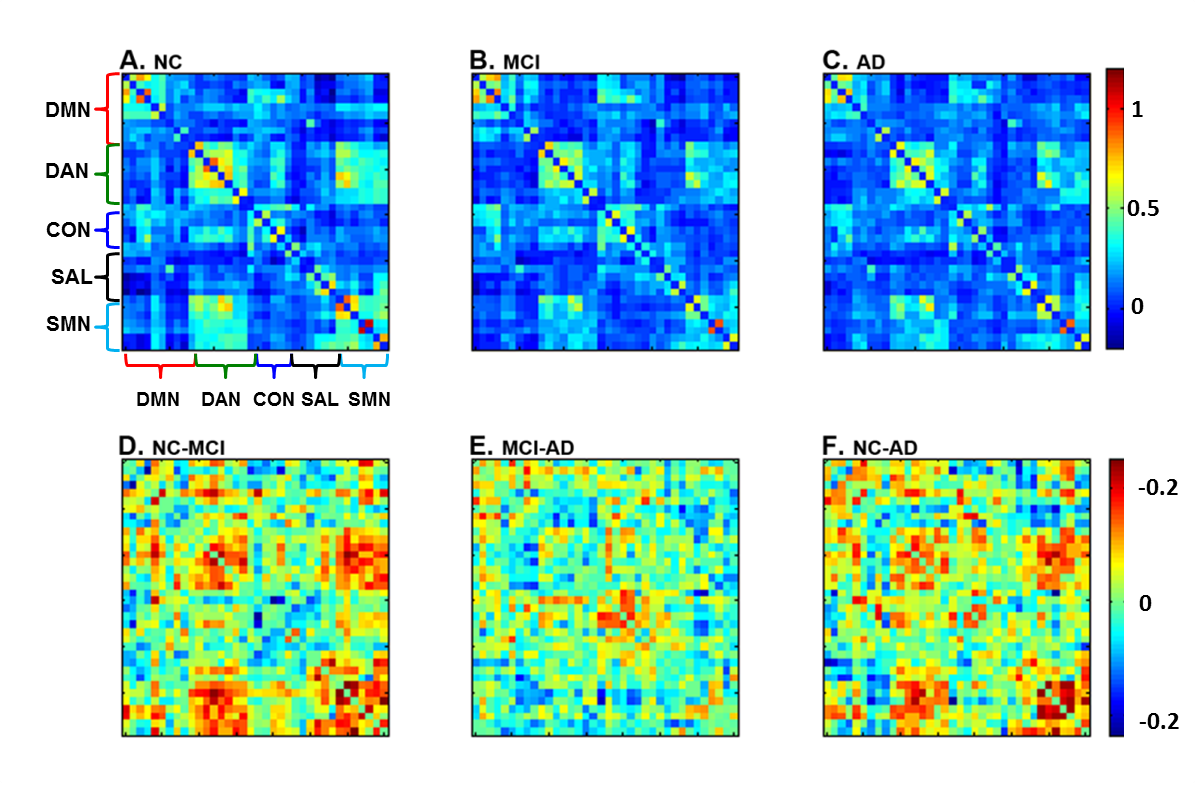


Figure S1. Mean absolute Z-score matrices for the three groups. Each figure shows a 36×36 square matrix in which each entry indicates the mean strength of the functional connectivity between each pair of brain regions. Intra-network correlations appear on diagonal blocks; inter-network correlations appear in off-diagonal blocks. (A) In the NC group, the majority of strong, positive functional connectivities were within each network, and most negative correlations were between different networks. (B-C) In the MCI group, the pattern was similar, but the strengths of the intra-network and inter-network connectivities were reduced compared with those in the NC group, and the correlations were even more reduced in the AD group. Figures D–F show the differences in mean correlation strength between the NC and MCI, NC and AD, and MCI and AD groups. (D) The NC-MCI difference matrix (NC minus MCI) shows that the signs of the correlation differences match the signs of the correlations themselves for a majority of ROI pairs. The warm hues predominantly indicate reduced correlations, whereas the cool hues predominantly indicate increased correlations. (E) The NC-AD difference matrix showing the larger correlation difference. (F) The MCI-AD difference matrix showing the smaller correlation difference than that between NC and MCI.

# Figure S2.


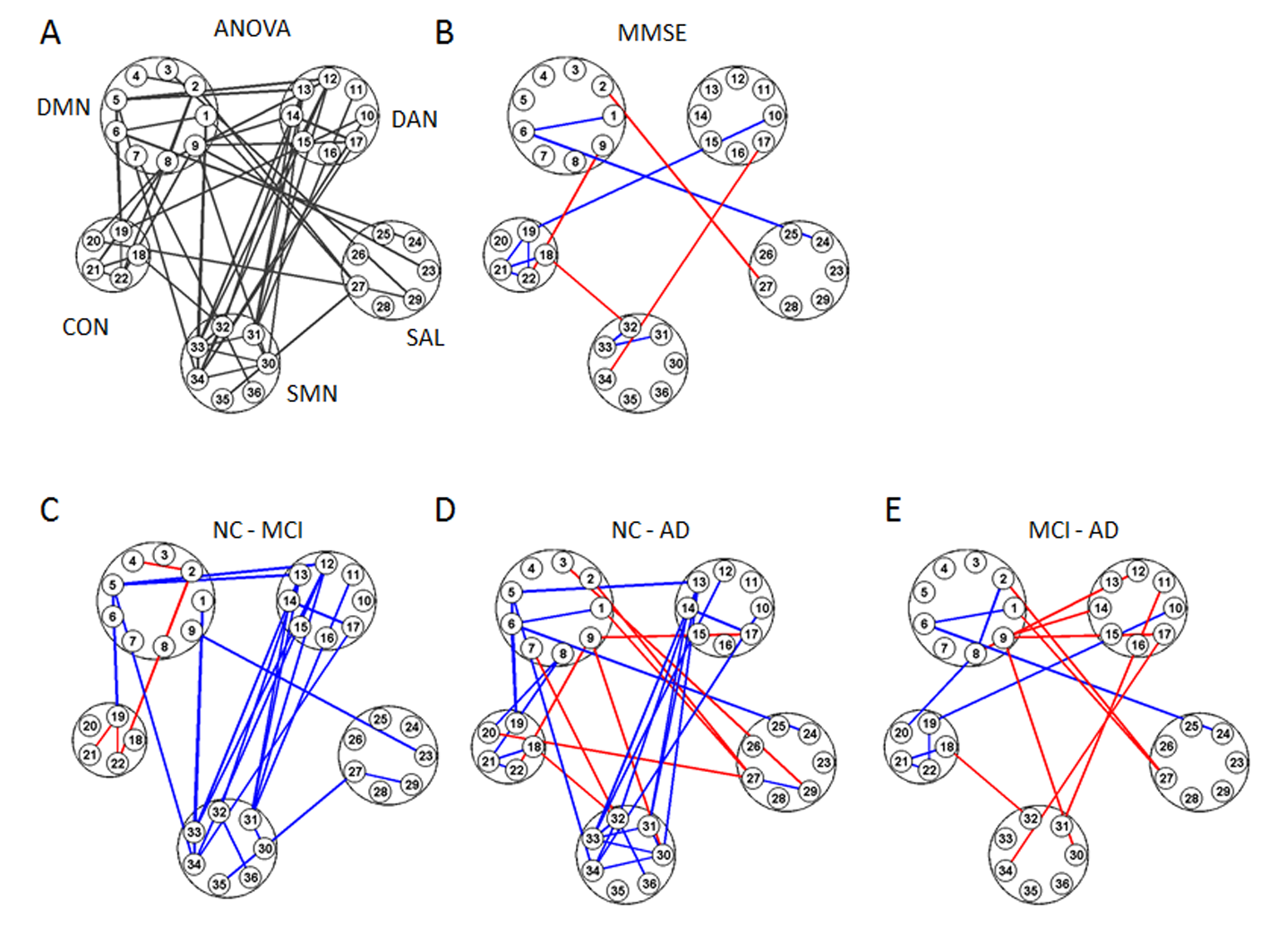


Figure S2 .(A) Distribution of the altered functional connectivity. All affected ROI pairs for all 5 RSNs, except for intra-network ROI pairs, were mainly distributed between the DMN and other RSNs and between the DAN and the SMN.(B) The correlations between the functional strength of the affected ROI pairs and the MMSE scores. The blue color represents the functional connectivity that shows positive correlations with the MMSE scores, and the red color represents negative correlations. (C) The differences in connectivity between the NC and MCI groups. (D) The differences in connectivity between the NC and AD groups. (E) The differences in connectivity between the MCI and AD groups. The blue color indicates that the functional connectivity of the former group is stronger than that for the latter, and the red color indicates the reverse.

.

# Reference

1. Wang P.*, et al.* Perceptual and response interference in Alzheimer's disease and mild cognitive impairment. *Clin Neurophysiol* **124**, 2389-2396 (2013).

2. Zhang Z.*, et al.* Altered spontaneous activity in Alzheimer's disease and mild cognitive impairment revealed by Regional Homogeneity. *Neuroimage* **59**, 1429-1440 (2012).

3. Yao H.*, et al.* Decreased functional connectivity of the amygdala in Alzheimer's disease revealed by resting-state fMRI. *Eur J Radiol* **82**, 1531-1538 (2013).

4. Zhou B.*, et al.* Impaired functional connectivity of the thalamus in alzheimer' s disease and mild cognitive impairment: a resting-state FMRI study. *Curr Alzheimer Res* **10**, 754-766 (2013).

5. Zhang Z.*, et al.* Altered functional connectivity of the marginal division in Alzheimer's disease. *Curr Alzheimer Res* **11**, 145-155 (2014).

6. Morris J.C. The Clinical Dementia Rating (CDR): current version and scoring rules. *Neurology* **43**, 2412-2414 (1993).

7. McKhann G.M.*, et al.* The diagnosis of dementia due to Alzheimer's disease: recommendations from the National Institute on Aging-Alzheimer's Association workgroups on diagnostic guidelines for Alzheimer's disease. *Alzheimers Dement* **7**, 263-269 (2011).

8. Albert M.S.*, et al.* The diagnosis of mild cognitive impairment due to Alzheimer's disease: recommendations from the National Institute on Aging-Alzheimer's Association workgroups on diagnostic guidelines for Alzheimer's disease. *Alzheimers Dement* **7**, 270-279 (2011).

9. Sperling R.A.*, et al.* Toward defining the preclinical stages of Alzheimer's disease: recommendations from the National Institute on Aging-Alzheimer's Association workgroups on diagnostic guidelines for Alzheimer's disease. *Alzheimers Dement* **7**, 280-292 (2011).

10. Petersen R.C.*, et al.* Mild cognitive impairment: clinical characterization and outcome. *Arch Neurol* **56**, 303-308 (1999).

11. Brier M.R.*, et al.* Loss of intranetwork and internetwork resting state functional connections with Alzheimer's disease progression. *J Neurosci* **32**, 8890-8899 (2012).
